# Defense mechanisms in immune-mediated diseases: a cross-sectional study focusing on Severe Allergic Asthma and Hymenoptera Venom Anaphylaxis patients

**DOI:** 10.3389/fpsyg.2025.1608335

**Published:** 2025-06-18

**Authors:** Gabriella Martino, Mariagrazia Di Giuseppe, Orlando Silvestro, Carmelo Mario Vicario, Concetto Mario Giorgianni, Paolo Ruggeri, Giorgio Sparacino, Maria Rosaria Juli, Peter Schwarz, Vittorio Lingiardi, Gianluca Lo Coco, Sebastiano Gangemi, Luisa Ricciardi

**Affiliations:** ^1^Department of Clinical and Experimental Medicine, University of Messina, Messina, Italy; ^2^Department of History, Humanities and Society, University of Rome Tor Vergata, Rome, Italy; ^3^Department of Health Sciences, University Magna Graecia of Catanzaro, Catanzaro, Italy; ^4^Department of Cognitive Science, Psychology, Education and Cultural Studies, University of Messina, Messina, Italy; ^5^Department of Biomedical and Dental Sciences and Morphofunctional Imaging, University of Messina, Messina, Italy; ^6^Course Degree in Medicine and Surgery, University of Messina, Messina, Italy; ^7^Department of Endocrinology, Centre for Cancer and Organ Diseases, Copenhagen University Hospital, Rigshospitalet, Copenhagen, Denmark; ^8^Department of Dynamic and Clinical Psychology and Health Studies, “Sapienza” University of Rome, Rome, Italy; ^9^Department of Psychology, Educational Science and Human Movement, University of Palermo, Palermo, Italy

**Keywords:** clinical psychology, chronic immune-mediated diseases, defense mechanisms, emotion regulation strategies, Severe Allergic Asthma, Hymenoptera venom allergy, DMRS-SR-30

## Abstract

**Background:**

Chronic immune-mediated diseases, such as Severe Allergic Asthma (SAA) and Hymenoptera Venom Anaphylaxis (HVA), significantly impact quality of life. Defense mechanisms, as implicit emotion-regulation strategies, shape an individual’s adaptation to chronic stressors. This cross-sectional study explored the relationship among defensive functioning, psychological symptoms, and perceived physical and mental health in patients with SAA and HVA.

**Methods:**

To explore the role of defensive functioning in perceived physical and mental health 34 patients with SAA and 32 with HVA were assessed with the Short-Form Health Survey, the Beck Depression Inventory, the Hamilton Anxiety Rating Scale, the Toronto Alexithymia Scale, and the Defense Mechanisms Rating Scales Self Report-30; between-group differences, and mediation analyses were performed.

**Results:**

Defensive functioning was positively associated with mental health and negatively related to depressive symptoms, anxiety and alexithymia. Males reported significantly higher physical and psychological health than females. Patients with SAA exhibited significantly higher defensive functioning but worse physical health than HVA patients. Mediation analysis revealed that defensive functioning correlated with disease type and physical health, accounting for 39% of the explained variances. Moreover, defensive functioning independently predicted mental health.

**Conclusion:**

This study highlights the influence of implicit emotional regulation on psychophysiological well-being in patients with chronic immune-mediated disorders. Despite reporting lower perceived physical health, patients with SAA exhibited higher defensive functioning, suggesting that chronic conditions may shape distinct psychological adaptation processes. These findings support the importance of defence mechanisms assessment to tailor psychological interventions promoting well-being in patients with chronic diseases.

## 1 Introduction

Increasing evidence shows that clinical-psychological features may contribute to patients’ ability to manage chronic diseases, leading to several health outcomes. Moreover, chronic diseases may deeply impact patients’ psychological and physical health, increasing the risk of developing psychological distress (Martino et al., 2023b; Galli et al., 2019; Liu et al., 2022; Conway et al., 2024; Ricciardi et al., 2024; Silvestro et al., 2025). Among chronic diseases, severe allergic asthma (SAA) and Hymenoptera venom allergy (HVA) represent significant clinical challenges for both patients and public health. Due to potentially fatal events related to SAA and HVA and their impact on daily life management, these diseases need more attention from the scientific community (Schiener et al., 2017; World Health Organization, 2019).

SAA affects approximately 5–10% of patients with asthma and is characterized by chronic inflammation, recurrent exacerbations and reduction in pulmonary function. Patients with SAA exhibit inadequate symptoms control, even with adherence to high-dose inhaled corticosteroids (ICS) and long-acting beta2 agonists (LABA) treatment, resulting in a higher risk of potentially fatal asthma crises (Bagnasco et al., 2021; Ricciardi et al., 2023).

Severe Allergic Asthma (SAA) is a persistent phenotype of asthma driven by immunoglobulin E (IgE)-mediated hypersensitivity to airborne allergens, including Dust Mites, animal dander, moulds, and pollens such as *Parietaria judaica*, which is prevalent in the Mediterrenean area (Chung et al., 2014; Liotta et al., 2023). Allergen exposure triggers a type 2 (T2) driven immune response, lymphocyte T helper 2 mediated, leading to eosinophilic inflammation, mast cell activation, and the release of mediators such as histamine, prostaglandins, and leukotrienes; this cascade exacerbates airway hyperreactivity, mucus overproduction, and bronchoconstriction, contributing to persistent respiratory symptoms and disease severity (Holgate et al., 2009; Liao et al., 2024). The difficulty in symptom control, along with sudden exacerbations, may contribute to increased vulnerability to psychopathologies, particularly anxiety and depression, which significantly impair Health-Related Quality of Life (HR-QoL) (Patella et al., 2022; Roche et al., 2022). Moreover, occupational exposure to environmental allergens, pollutants, or triggers has been associated with the onset and the worsening of asthma symptoms, with outdoor workers at risk due to their higher levels of exposure (Giorgianni et al., 2024; Suarthana et al., 2024).

HVA is an IgE-mediated hypersensitivity condition triggered by occasional stings from bees or wasps, causing systemic reactions such as urticaria, angioedema, or sudden anaphylaxis, sometimes still reported as fatal (Sturm et al., 2018). In adults, the prevalence of systemic reactions among individuals with HVA varies between 0.5 and 3.3% in the USA and from 0.3 to 7.5% in Europe (Biló et al., 2005; Golden, 2005). Notably, 48.2% of severe hypersensitivity reactions in adults (aged >18 years) are attributed to Hymenoptera stings (Worm et al., 2014). Fear of re-stings, coupled with the unpredictable nature of anaphylaxis, can lead to significant psychological distress, including anxiety, social isolation, and diminished HR-QoL (Schoeben et al., 2020; Höfer et al., 2023). Outdoor workers, due to their heightened exposure to Hymenoptera species, are at an elevated risk of HVA-related incidents, underscoring the importance of preventive measures and education on proper sting management (Toletone et al., 2017).

In chronic diseases, there is a growing recognition of the link between psychological distress and immune system alterations mediated by neuroinflammatory processes highlighting the critical role of clinical psychological factors (Dantzer, 2018; Murdaca et al., 2022; Ravi et al., 2021). Evidence suggests a bidirectional relationship between chronic inflammatory processes and the increased risk of the onset of psychopathologies (Antunes et al., 2022). Liu et al. (2023) reported that following an asthma diagnosis, the likelihood of developing various subsequent mental disorders, such as mood disorders or personality disorders, was significantly elevated. The risk of developing asthma has also been reported after a prior mental disorder diagnosis and variations were observed across different psychopathologies. Therefore, the assessment of psychological functioning in patients with chronic immune-mediated diseases provides valuable insights into illness adaptation processes, including SAA and HVA (Livneh, 2022; Martino et al., 2023a; Silvestro et al., 2024).

Chronic diseases are stressful life conditions that require a constant compromise between the demands of daily life and the requirements of care (Berg and Upchurch, 2007). Psychological factors, including emotion regulation strategies, coping mechanisms, alexithymia, and personality traits, have been acknowledged as crucial in determining physical and mental well-being in both healthy (Balzarotti et al., 2016; Saxena et al., 2011; Tanzilli et al., 2024) or not populations (Silvestro et al., 2023; Martino et al., 2020b, 2023a). Among adjustment variables in chronic immune-mediated diseases, alexithymia has particular interest. Alexithymia is defined as difficulty in identifying and describing emotions, leading to impaired emotional awareness and limited imaginative processes (Nemiah and Sifneos, 1970; Bagby et al., 1994a,b). Several studies have shown that alexithymia is associated with dysregulation of the hypothalamic–pituitary–adrenal axis, resulting in elevated cortisol and pro-inflammatory cytokines, which may amplify inflammation in chronic immune conditions (Goerlich and Votinov, 2023; Guilbaud, 2025). Concerning SAA and HVA, patients with clinically significant alexithymia scores reported heightened psychological distress and poorer perceived mental and social well-being (Ricciardi et al., 2024). Few studies have also linked alexithymia to altered perception of bodily sensations, reduced treatment adherence, and greater vulnerability to psychological distress (Lumley et al., 1996; Xu et al., 2024; Silvestro et al., 2025). These findings suggest the relevant role of alexithymia in psychological assessment of patients with SAA and HVA, since its impact on emotional regulation and stress responses may substantially influence disease management and outcomes.

Within the biopsychosocial framework (Sergi, 2024), these observations emphasize the key role of psychological factors, such as alexithymia and emotion regulation strategies, in the aetiology and progression of various chronic diseases, including gastrointestinal (Martino et al., 2020a, 2023b; Naude et al., 2025; Yan et al., 2023), dermatological (Biazus Soares et al., 2024; Goyal and Prabhu, 2023; Polaskey and Chovatiya, 2025), and cardiovascular disorders (Di Giacomo et al., 2024; Nation et al., 2022; Spatola et al., 2024).

Defense mechanisms are relevant for the psychological well-being of chronic patients as they contribute to stress management (Békés et al., 2023). The interest in defense mechanisms and their association with the progression of chronic diseases has recently increased in scientific debates as an essential aspect of implicit emotional regulation. In immune-mediated diseases, psychological processes that infer infection risk from perceptual cues, and that respond to these perceptual cues through the activation of aversive emotions, cognitions and behavioral impulses, are extremely important (Schaller, 2011). Research has demonstrated that acute and chronic psychological stress can induce pronounced changes in innate and adaptive immune responses and that these changes are predominantly mediated via neuroendocrine mediators from the hypothalamic–pituitary–adrenal axis and the sympathetic-adrenal axis (Kemeny and Schedlowski, 2007). With regard to psychological defense mechanisms, few studies have found that mature defenses promote lower disease progression in patients diagnosed with gastrointestinal cancer, while immature defense mechanisms may contribute to more advanced disease stages (Moazed et al., 2024). Moreover, there is evidence of the mediating role of defense mechanisms on psychological distress assessed in various clinical samples, including patients affected by chronic diseases (Zerach and Elklit, 2020; Berk and Baykara, 2020; Musetti et al., 2023; Romeo et al., 2022). From the best of our knowledge, no studies have investigated the role of defense mechanisms in chronic immune-mediated diseases, such as SAA and HVA, which would potentially be a relevant aspect of emotion regulation impacting on the disease that need an in-depth investigation.

In the present cross-sectional study, we aimed to investigate the role of defensive functioning in protecting individuals with chronic immune-mediated diseases against physical and psychological perceived distress. The study aimed to examine whether: (1) higher defensive functioning is associated with higher physical and mental well-being, lower depression, anxiety, and alexithymia; (2) patients with SAA, although experience higher physical and psychological distress than subjects with HVA, show higher defensive functioning than patients with HVA; (3) physical and psychological well-being is mediated by defensive functioning. The expectations were: (1) that defensive functioning would be positively related to physical and mental health and, conversely, negatively related to psychological symptoms of depression, anxiety and alexithymia; (2) to observe higher use of mature defenses in patients with SAA compared to controls with HVA; (3) to find that defensive functioning would mediate both physical and psychological well-being.

## 2 Methods

### 2.1 Participants

A cross-sectional study was conducted including 66 patients with chronic immune-mediated diseases, 34 with a diagnosis of SAA and 32 HVA.

Participants attended the Allergy and Clinical Immunology Outpatient Clinic in the Department of Clinical and Experimental Medicine of the University Hospital of Messina, Italy. Patients in the SAA group were managed with Omalizumab, a biologic targeting IgE, in combination with high-dose ICS + LABA. Conversely, patients with HVA underwent venom immunotherapy (VIT) tailored to their sensitisation profiles. Nine patients received VIT with bee venom, while 23 were treated with wasp venom. All patients were enrolled during the maintenance phase of VIT, consisting of 100 μg of venom extract, administered subcutaneously every 4 to 6 weeks. Patients were enrolled during their first year on the VIT maintenance dose. None of the patients were receiving concomitant treatments or medications that could influence mood or anxiety, such as H1-antihistamines or systemic corticosteroids, during enrolment.

Eligibility criteria excluded patients with age <18 years old and suffering from cognitive impairment, moderate to severe renal or hepatic failure, heart failure classified as NYHA class ≥2, malignancies, malabsorption disorders, thyroid, parathyroid, or adrenal dysfunction, as well as individuals with psychopathologies or history of psychotropic drug assumption.

The cohort consisted of 34 males (51.5%, of which 16.7% with SAA and 34.8% with HVA) and 32 females (48.5%, of which 34.8% with SAA and 13.7% with HVA), with an average age of 51.7 years (SD = 15.0; range: 20–78 years). Regarding educational attainment, one participant had completed elementary school (1.5%), 14 middle school (21.2%), 40 high school (60.6%), 10 had a university degree (15.2%), and 1 reported other qualifications (1.5%). Employment and marital status varied; 45 participants were employed (68.2%), 5 unemployed (7.6%), 11 retired (16.7%), and 5 homemakers (7.6%). 47 were married or in a relationship (71.2%), 14 were single (21.2%), 3 divorced (4.5%), and 2 widowed (3.0%).

### 2.2 Measures

#### 2.2.1 The Beck Depression Inventory-II (BDI-II)

BDI-II is a self-report questionnaire comprising 21 items rated on a four-point Likert scale (Beck et al., 1996; Ghisi et al., 2006), evaluating depressive symptoms’ presence and severity. BDI-II presents a bifactorial structure, assessing both neurovegetative features (e.g., changes in appetite, irritability, loss of energy) and cognitive facets of depression (e.g., sadness, guilty feelings, punishment feelings). For the present study, the Italian version of the BDI-II was used, considering the total score to assume depressive symptoms. The instrument has been validated in Italy and demonstrates satisfactory psychometric properties in both healthy and clinical populations, with Cronbach’s *α* = 0.84 (Montano and Flebus, 2006; Maggi et al., 2023).

#### 2.2.2 The Hamilton Anxiety Rating Scale (HAM-A)

Anxiety symptoms’ levels were assessed using the HAM-A, a self-administered instrument comprising 14 items rated on a Likert scale from 0 to 4 (Hamilton, 1959). The scale evaluates somatic (e.g., cardiac, sensory, respiratory issues) and psychological symptoms (e.g., insomnia, intellectual, and low mood). In the present study, the Italian version of the HAM-A was administered. This version is widely used in Italy and has been employed in previous studies, demonstrating consistent applicability (Martino et al., 2021a,b).

#### 2.2.3 The Short-Form Health Survey-36 (SF-36)

HR-QoL was assessed using the Italian adaptation of SF-36. This self-administered questionnaire measures eight dimensions of HR-QoL: physical functioning, physical role limitations, bodily pain, general health, vitality, mental health, emotional role limitations, and social functioning (Ware and Sherbourne, 1992). Scores range from 0 to 100, with higher values indicating better HR-QoL and lower values reflecting a decline in HR-QoL. The psychometric properties of the Italian version have been standardized, with Cronbach’s *α* coefficients between 0.77 and 0.93 for the eight dimensions (Apolone and Mosconi, 1998). Consistent with the study objectives, the present study focused solely on the physical and mental health indices.

#### 2.2.4 The Toronto Alexithymia Scale-20 (TAS-20)

Alexithymia was evaluated through TAS-20, a 20-item self-report questionnaire scored on a five-point Likert scale (Taylor et al., 1997; Taylor, 2000; Bagby et al., 1994a; Bagby et al., 1994b). The scale identifies alexithymia profiles based on three cut-off scores: ≥61 (alexithymic), 51–60 (borderline), and ≤50 (non-alexithymic). The TAS-20 assesses three core dimensions of alexithymia: difficulty identifying feelings (DIF), difficulty describing feelings (DDF), and externally oriented thinking (EOT). For this study, the Italian version of the TAS-20 was employed, demonstrating robust psychometric reliability, with a Cronbach’s α of 0.86 for the total score and similarly strong values ranging from 0.79 to 0.86 for the three subscales: DIF, DDF, and EOT (Bressi et al., 1996).

#### 2.2.5 The Defense Mechanisms Rating Scales—Self Report—30 (DMRS-SR-30)

The DMRS-SR-30 (Di Giuseppe et al., 2020) is a 30-item self-reported questionnaire assessing individuals’ defensive functioning as described in the gold standard DMRS theory (Perry, 1990, 2014) The DMRS-SR-30 items were extracted from the Q-sort version of the DMRS and adapted for the self-report version (DMRS-Q; Di Giuseppe, 2024). The DMRS-SR-30 is comprehensive of the following quantitative scores: (1) a continuous 7-point index of Overall Defensive Functioning (ODF), with lower scores indicating lower defensive maturity and, conversely, higher scores indicating higher defensive maturity; (2) a proportional score of three defense categories (i.e., mature defenses, neurotic defenses, immature defenses); (3) a proportional score of seven hierarchically ordered defense levels (i.e., high-adaptive defense level, obsessional defense level, neurotic defense level, minor image-distorting defense level, disavowal defense level, higher image-distorting defense level, and action defense level); and (4) a proportional score for each of the 28 defense mechanisms included in the hierarchy. Preliminary validation studies demonstrated good psychometric properties of the DMRS-SR-30 such as good reliability for ODF and defensive categories (ICC ranging from 0.68 to 0.89), good criterion, concurrent, convergent and discriminant validity (Di Giuseppe et al., 2020), excellent internal consistency of the DMRS-SR-30 three-factors model (Prout et al., 2022).

### 2.3 Procedure

Participants were recruited at the Allergy and Clinical Immunology Outpatient Clinic in the Department of Clinical and Experimental Medicine of the University Hospital of Messina, Italy, between January and May 2022. All participants provided written informed consent before inclusion in the study. They were informed about the research objectives, privacy protections, and the use of anonymous data in compliance with the European General Data Protection Regulation (GDPR) 2016/679. The study was approved by the Ethics Committee of the University Hospital of Messina (Protocol number 16/19) and adhered to the ethical principles outlined in the Declaration of Helsinki (2013). The administration of assessment tools was conducted during a gold-standard clinical interview performed by a clinical psychology researcher. This procedure ensured the quality and reliability of the information gathered.

### 2.4 Statistical analyses

To test whether higher defensive functioning is associated with higher physical and mental well-being, lower depression, anxiety, and alexithymia, we performed a Pearson Correlations analysis to compare the ODF to physical health, mental health, depression, anxiety, and alexithymia. An independent sample *t*-test was executed to evaluate whether patients with SAA show higher defensive functioning than patients with HVA despite their higher physical and psychological distress. To evaluate whether physical and psychological well-being is mediated by defensive functioning, we used the General Linear Model (GLM) Mediation Analysis. All analyses were performed with the statistical software Jamovi Version 2.4.

## 3 Results

### 3.1 Descriptive statistics

The study involved 66 participants divided into two groups: the group with SAA included 34 participants, of which the majority were females (*N* = 23; 68%), while the group with HVA included 32 participants, of which the majority were males (*N* = 23; 72%). Descriptive statistics are reported in Table 1.

**Table 1:** Descriptive statistics.

	Severe allergic asthma (male, *n* = 11)	Severe allergic asthma (female, *n* = 23)	Hymenoptera venom allergy (male, *n* = 23)	Hymenoptera venom allergy (female, *n* = 9)
Defensive Function (Mean ± SD)	5.29 ± 0.60 (4.30–6.32)	5.04 ± 0.58 (4.48–6.68)	4.85 ± 0.45 (3.97–5.70)	4.74 ± 0.30 (4.17–5.22)
Physical Health (Mean ± SD)	54.09 ± 20.72 (15–85)	40.22 ± 20.97 (15–90)	70 ± 18.83 (20–95)	61.67 ± 27.04 (30–100)
Mental Health (Mean ± SD)	64 ± 14.64 (44–92)	55.3 ± 15.24 (28–84)	64.57 ± 19.91 (20–88)	53.78 ± 21.92 (2–20)
Depressive Symptoms (Mean ± SD)	12.09 ± 5.96 (3–21)	14.13 ± 5.88 (4–25)	10.52 ± 6.27 (3–29)	11.22 ± 6.94 (2–20)
Anxiety symptoms (Mean ± SD)	24.64 ± 10.31 (7–36)	29.52 ± 10.77 (5–49)	24.74 ± 9.17 (7–41)	28.11 ± 13.47 (5–44)
Alexithymia (Mean ± SD)	54.00 ± 14.62 (29–75)	55.65 ± 12.97 (33–77)	61.74 ± 13.51 (35–89)	54.44 ± 9.24 (38–65)

All values represent *M* ± SD (minimum – maximum). Higher scores indicate better physical and mental health, whereas higher scores for depressive symptoms, anxiety symptoms, and alexithymia indicate higher severity.

The mean ODF among the whole sample fell in the neurotic range (*X* = 4.97; SD = 0.53), with slightly higher scores observed in participants with SAA. Physical and mental health scores reported by our sample were 55.83 and 59.77 on average, respectively. Females reported lower scores than males on both indexes of health, although physical health was generally lower in the SAA. Depression, anxiety and alexithymia mean scores assessed in the whole sample were 12.14, 26.85, and 57.33, respectively. SAA group reported higher depression, whereas females reported higher anxiety. Alexithymia above the average emerged only for male participants with HVA.

### 3.2 Association between defensive functioning, physical and mental health, and psychological symptoms

Pearson correlations between defensive functioning, physical and mental health, and psychological symptoms are reported in Table 2.

**Table 2:** Pearson correlations between defensive functioning, health, and symptoms.

	Physical health	Mental health	Depressive symptoms	Anxiety symptoms	Alexithymia
Defensive functioning	0.216	0.316^*^	−0.329^**^	−0.287^*^	−0.381^**^
Physical health	–	0.605^***^	−0.541^***^	−0.524^***^	−0.114
Mental health	–	–	−0.646^***^	−0.387^**^	−0.228
Depressive symptoms	–	–	–	0.589^***^	0.160
Anxiety symptoms	-	–	–	–	0.129

^*^p < 0.05; ^**^p < 0.01; ^***^p < 0.001.

In our sample of participants with chronic immune-mediated diseases, defensive functioning was significantly associated with mental health (*r* = 0.316; *p* = 0.010). Positive correlations were also found with physical health, although not reaching the level of statistical significance. Conversely, the ODF was negatively associated with psychological symptoms of depression and anxiety, as well as with alexithymia traits. In particular, the use of mature defenses was found significantly negatively correlated with depression (*r* = −0.339; *p* = 0.005), anxiety (*r* = −0.281; *p* = 0.022), and alexithymia (*r* = −0.474; *p* < 0.001) while the use of immature defenses was found positively correlated with psychological symptoms [i.e., depression *r* = 0.302; *p* = 0.014; anxiety (*r* = 0.265; *p* = 0.032); alexithymia (*r* = 0.319; *p* = 0.009)].

### 3.3 Differences in defensive functioning, physical and mental health, and psychological symptoms among SAA versus HVA patients

A preliminary mean comparison analysis was performed to check gender differences among analyzed variables. The results of the independent sample *t*-tests are displayed in Table 3 and showed that males reported significantly higher physical and mental health defensive functioning than females, with Cohen’s d effect size of 0.82 and 0.54, respectively. No significant gender differences were found for defensive functioning and psychological symptoms.

**Table 3:** Gender comparison among defensive functioning, health, and symptoms.

	*t*	df	*p*	Effect size (d)
Defensive functioning	0.223	64	0.824	0.055
Physical health	3.353	64	0.001^**^	0.826
Mental health	2.193	64	0.032^*^	0.540
Depressive symptoms	−1.501	64	0.138	−0.370
Anxiety symptoms	−1.725	64	0.089	−0.425
Alexithymia	1.215	64	0.229	0.299

*t*, Student’s *t*-test statistic; df, degrees of freedom; d, Cohen’s d; ^*^p < 0.05; ^**^p < 0.01; ^***^p < 0.001.

Independent sample *t*-tests were performed to analyze differences between groups with different chronic immune-mediated diseases (Table 4).

**Table 4:** Group comparison among defensive functioning, health, and symptoms.

	*t*	df	*p*	Effect size (d)
Defensive functioning	2.435	64	0.018^*^	0.660
Physical health	−4.340	64	< 0.001^***^	−1.069
Mental health	−0.763^a^	64	0.448	−0.188
Depressive symptoms	1.824	64	0.073	0.449
Anxiety symptoms	0.865	64	0.390	0.213
Alexithymia	−1.422	64	0.160	−0.350

*t*, Student’s *t*-test statistic; df, degrees of freedom; d, Cohen’s d; ^*^p < 0.05; ^**^p < 0.01; ^***^p < 0.001.

Participants with SAA reported greater physical discomfort than participants with HVA. At the same time, the group with SAA reported significantly higher defensive functioning than the group with HVA. No statistically significant differences between groups were found for mental health, depression, anxiety, and alexithymia.

### 3.4 Mediation effect of defensive functioning on physical and mental well-being

**Figure 1:**
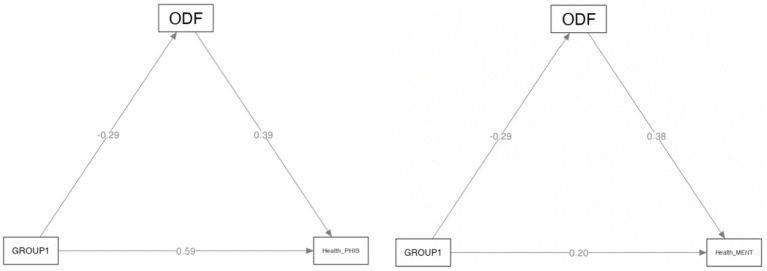
The path model of mediation effect of defensive functioning on physical and mental health. ODF, Overall Defensive Functioning. Categorial independent variables (factors) are represented by contrast indicators. For variable GROUP, the contrasts are GROUP1 = 2–1.

The path models analyzed in GLM mediation analyses are displayed in Figure 1.

Direct, indirect (mediation) and total effects of chronic immune-mediated diseases and ODF on physical health are displayed in Table 5, while direct, indirect (mediation) and total effects of chronic immune-mediated diseases and ODF on mental health are displayed in Table 6.

**Table 5:** Mediation effect of defensive functioning on physical health.

Effect type	Pathway	Estimate	SE	95% C.I.		*Β*	*z*	*p*
				Upper	Lower			
Indirect	IMD → ODF → physical health	−5.434	2.626	−10.579	−0.288	−0.113	−2.07	0.038^*^
Component	IMD → ODF	−0.307	0.124	−0.550	−0.064	−0.291	−2.47	0.013^*^
	ODF → physical health	17.714	4.683	8.535	26.896	0.388	3.78	< 0.001^***^
Direct	IMD → physical health	28.384	4.934	18.713	38.054	0.590	5.75	< 0.001^***^
Total	IMD → physical health	22.950	5.247	12.667	33.233	0.477	4.37	< 0.001^***^

IMD, Immune-mediated diseases; ODF, Overall Defensive Functioning; Estimate, unstandardized regression coefficient; SE, standard error; C.I., confidence interval (computed using the Standard Delta Method); *β*, completely standardized effect size; *z*, test statistic from the normal distribution; ^*^p < 0.05; ^**^p < 0.01; ^***^p < 0.001.

**Table 6:** Mediation effect of defensive functioning on mental health.

Effect type	Pathway	Estimate	SE	95% C.I.		Β	*z*	*p*
				Upper	Lower			
Indirect	IMD → ODF → mental health	−3.933	2.024	−7.333	0.033	−0.109	−1.946	0.052
Component	IMD → ODF	−0.307	0.124	−0.590	−0.036	−0.291	−2.472	0.013^*^
	ODF → mental health	12.822	4.078	4.829	20.815	0.376	3.144	0.002^**^
Direct	IMD → mental health	7.347	4.296	−1.074	15.767	0.204	1.710	0.087
Total	IMD → mental health	3.414	4.441	−5.291	12.117	0.095	0.769	0.442

IMD, Immune-mediated diseases; ODF, Overall Defensive Functioning; Estimate, unstandardized regression coefficient; SE, standard error; C.I., onfidence interval (computed using the Standard Delta Method); *β*, completely standardized effect size; *z*, test statistic from the normal distribution; ^*^p < 0.05; ^**^p < 0.01; ^***^p < 0.001.

The direct effect of the chronic immune-mediated diseases on physical health was significant (*p* < 0.001) and corresponded to an increase of 0.59 standard deviations (SD) on the physical health scale for the group with HVA as compared to the group with SAA. The mediating effect of defensive functioning on physical health resulted further significant (*p* = 0.038) and contributed to improving physical health by 0.39 SD for the group with HVA as compared to the group with SAA. However, the group with HVA also showed a significantly lower ODF than the group with SAA (*p* = 0.013), resulting in a total effect of 0.48 SD respiratory disease on physical health mediated by ODF.

The direct effect of chronic immune-mediated diseases on mental health was not significant (*p* < 0.087), as well as the mediating effect (indirect) of ODF on mental health (*p* = 0.052). Accordingly, the total effect of chronic immune-mediated diseases on mental health mediated by ODF was also not significant. Only the direct effect of ODF on mental health was significant (*p* = 0.002), corresponding to an increase of 0.38 *SD* on the mental health scale for each SD increase in ODF.

## 4 Discussion

The present study represents an original empirical contribution to the knowledge of the impact of implicit emotional regulation on the psychological well-being of patients with chronic immune-mediated diseases. The study provides research advances on the under-investigated role of defensive functioning as a mediator of psychological symptoms, such as depression, anxiety, and alexithymia, in chronic patients. It also offers clues to the scientific debate on the importance of observing and assessing implicit aspects of psychological functioning that could contribute to the onset, course and treatment of chronic diseases.

Our first hypothesis, that defensive functioning in our sample was positively related to physical and mental health and, conversely, negatively related to psychological symptoms of depression, anxiety and alexithymia, was fully confirmed. Higher ODF, which indicates greater defensive maturity, corresponding to a better ability to manage the emotional distress caused by internal conflict and external stressful situations, was associated with higher mental health and lower depression, anxiety, as well as with alexithymia. Associations between ODF and physical health were also found in the expected direction, although not reach the level of statistical significance in our sample. As expected, higher use of immature defenses was found in participants that reported greater psychological symptoms of depression, anxiety, and alexithymia, indicating that poor implicit emotional regulation hindered the management of life difficulties, including the experience of being chronically ill. Conversely, patients who were able to resort to greater use of mature defenses showed better implicit emotional regulation capacities that protected them from psychological distress. According to recent findings, mature defenses constitute essential psychological resources that should be considered in tailoring clinical intervention (Blanco et al., 2023; Vaillant, 2000). Through therapeutic interventions aimed at modifying immature defenses into more mature and adaptive defenses, it is possible to equipe patients with more effective implicit emotional regulation strategies for managing chronic illness and its psychological correlates.

Our second hypothesis, that patients with SAA, despite greater physical and psychological distress, could show higher defensive functioning than patients with HVA, was partially confirmed. Patients with SAA, who showed greater physical discomfort than patients with HVA, reported significantly higher defensive functioning with no differences between groups on mental health, depression, anxiety, and alexithymia. These findings suggest interesting reflections about how chronic diseases could lead to a decline in defensive functioning. Research has shown that physical and psychological vulnerability is often associated with the use of more immature defense mechanisms (Di Giuseppe et al., 2024a; Kreitler, 2004). However, few studies found that aggravators of illness are associated with more stable defensive functioning (Heim et al., 1978) and that mature defenses are associated with higher survival probability in cancer patients (Beresford et al., 2006). In an evolutionary perspective, the need to deal with disadvantaged conditions (socio-economic, cultural, physical, psychological, etc.) can activate individual resiliency, which is driven by a mature and adaptive defensive functioning. Although these are just preliminary findings that should be carefully interpreted, our results suggest that patients with SAA might have learned by necessity to manage their chronic illness compared to HVA patients by using more adaptive defenses. Conversely, HVA patients, who are occasionally victims of Hymenoptera stings, did not necessarily need to develop effective emotional regulation strategies to counteract the physical effects of their disease.

Finally, our third hypothesis, that physical and psychological well-being is mediated by defensive functioning, was also partially confirmed. Although we expected that in our sample of individuals with SAA, defensive functioning would mediate both physical and psychological well-being, results showed that the mediating effect of defensive functioning significantly correlated to physical health but not to mental health. However, the direct effect of ODF on both physical and mental health was found to be significant and corresponded to a consistent increase in psychological well-being. To understand these findings, it is important to highlight that SAA patients showed greater physical distress and higher ODF than HVA patients. The direct effect of chronic immune-mediated diseases had an impact on the perception of physical distress, but since SAA patients had a significantly higher ODF, the mediation effect of ODF was able to counterbalance the higher physical distress in the more impaired SAA patients. Our findings on the impact of ODF on psychological well-being support previously reported data (Zimmerman et al., 2019). Furthermore, these findings confirm previous literature on the strong relationship between implicit emotion regulation, assessed as defense mechanism, and psychological well-being (Di Giuseppe et al., 2022; Bincoletto et al., 2025). They also suggest a potential relationship between implicit emotional regulation and physical health that needs further investigation to be understood in depth. While it is well known that defense mechanisms play a fundamental role in psychological functioning, researchers have recently focused on their studies on defenses as a factor that can influence physical well-being. Although controversial, research findings increasingly suggested considering psychological factors such as implicit emotional regulation as essential in the understanding of chronic diseases and, therefore, in treatment planning (Di Giuseppe et al., 2024b).

Despite its potential, this study has some limitations. Firstly, the small sample does not allow us to generalize results to the broader population of patients with chronic immune-mediated diseases; further investigations into larger case–control samples should be carried out to confirm these findings. Secondly, the unequal proportion of women and men in the two groups of SAA and HVA might have slightly biased the results since, generally, women show lower defensive functioning than men. Future studies should equally distribute gender across groups to avoid a possible overrepresentation of some gender-associated defenses. Third, the cross-sectional research design did not allow inferences about the causal relations among defense mechanisms and other studied variables. Prospective longitudinal studies might inform on causal long-term effects of defensive functioning on physical and psychological functioning. Finally, the use of self-reported questionnaires to assess psychological variables might have determined response biases typical of self-assessment measures. Further research using observer-rated instruments on defenses will be needed to test the generalizability of our results.

## 5 Conclusion

The systematic investigation of defense mechanisms in the clinical follow-up of patients with chronic diseases could allow us to obtain essential information on the impact of implicit emotional regulation on psychological functioning. Our cross-sectional study on patients with chronic immune-mediated diseases such as SAA and HVA highlights that in these patients, the interrelation between physical and mental well-being has an important impact on their health. The assessment of defenses can be achieved through the application of valid and reliable measures, such as the Defense Mechanisms Rating Scales Self-Report-30 instrument. Research advances on the under-investigated role of defensive functioning as a mediator of psychological symptoms, such as depression, anxiety, and alexithymia, in chronic patients can offer clues to the scientific debate on the importance of observing and assessing implicit aspects of psychological functioning on the onset, course and treatment of chronic disease.

## Data Availability

The raw data supporting the conclusions of this article will be made available by the authors, without undue reservation.
